# A unifying theory of ITD-based sound azimuth localization at the behavioral and neural levels

**DOI:** 10.1186/1471-2202-14-S1-P39

**Published:** 2013-07-08

**Authors:** Victor Benichoux, Marcel Stimberg, Bertrand Fontaine, Romain Brette

**Affiliations:** 1 Equipe Audition, Département d'Etudes Cognitives, Ecole Normale Supérieure, Paris, 75005, France; 2 Laboratoire Psychologie de la Perception, CNRS and Université Paris Descartes, Paris, 75006, France; 3 Dominick P. Purpura Department of Neuroscience, Albert Einstein College of Medicine, Bronx, New York, USA

## 

In many species, azimuthal sound source localization relies on the processing of fine temporal differences between the incoming signals at both ears (interaural time differences, ITDs). There exists no consensual theory of ITD-based localization that explains the behavioral and neural data alike. The classical view of a place code for localization [1] is questioned by electrophysiological data [2], while its alternative is functionally inefficient [3]. We propose as a functional principle that the system performs a maximum-likelihood estimation of the position of the source given the cues in the stimulus. This Bayesian approach implies that the behavioral and neural data are constrained by natural distributions of binaural cues, as observed in acoustical recordings of head related transfer functions (HRTFs). We first record and analyze HRTFs in humans and cats. Then we discuss the implications of our hypothesis on psychoacoustical data in humans and electrophysiological data in the cat.

In a maximum-likelihood approach, the current observed cue is compared to the a priori distribution of cues (marginal prior normalization). It is thus fundamental to uncover what the cues are and how they are distributed across the spectrum. We recorded HRTFs in different species, and performed simulations of natural environments to quantify the robustness of ITD cues. We find that ITD is a frequency-dependent quantity that decreases by about 30% across the spectrum, and that such variations occur within the bandwidth of a cochlear filter. We also show how the distributions of cues vary across frequencies, in relation with various features of the environment such as reflections.

Because the ITD as a constant delay is an insufficient cue, azimuth should be extracted by the system based on a frequency-dependent representation of ITD. We test this prediction in a psychoacoustical setup. Using a matching paradigm, subjects are asked to adjust the lateralization of two noises with different frequency contents, by varying the ITD of one of the stimuli. The HRTF data allows us to predict that the higher frequency sounds should be matched with a lower ITD than the lower frequency sound. We show how this prediction is met both qualitatively and quantitatively in our experiment.

We give a model of the function of binaural cells in the cat brainstem. We predict the responses of those neurons to binaural beats at different frequencies from the cat HRTFs. We show how this simple model can explain already observed features of the electrophysiological literature [4], namely the presence of cells sensitive to frequency-dependent interaural delays.

Finally, we propose a spiking neuron implementation of this maximum-likelihood principle. Cells are tuned to the frequency-dependent cues of their best position by means of both cochlear mismatches and axonal delays [5]. The Bayesian marginal prior normalization is implemented through the use of inhibition. Probing the model with various input sources, in a simulated virtual environment, we show that the network accurately localizes sound sources, comparably with an optimal Bayesian observer. Moreover, this model predicts qualitative differences in those observations for mammals of different sizes such as the cat and gerbil.
